# Dataset on green macroprudential regulations and instruments: Objectives, implementation and geographical diffusion

**DOI:** 10.1016/j.dib.2019.103870

**Published:** 2019-03-21

**Authors:** Paola D'Orazio, Lilit Popoyan

**Affiliations:** a Chair of Macroeconomics and Research Department Closed Carbon Cycle Economy, Ruhr Universität Bochum, Germany; b Institute of Economics, Scuola Superiore Sant’Anna, Italy

**Keywords:** Climate change, Climate finance gap, Banking regulation, Macroprudential policy, Central banking

## Abstract

The dataset presented in this article contains the data gathered when surveying existing “green” macroprudential regulations and instruments. Official central banks’ documents and acts, as well as financial institutions reports, have been considered. In particular, for the selected OECD and non-OECD countries, the dataset contains information on the type of institutional governance, the mandate, the type of green regulation, the institution responsible for its implementation or promotion, the official reference and the related link to the document, as of February 2019. The dataset is made publicly available considering the actively evolving debate about the adoption of green prudential requirements and the implementation of green prudential instruments.

**Table:** Specifications table

Subject area	*Economics*
More specific subject area	*Macroprudential regulation, Green finance*
Type of data	*Text, Excel dataset*
How data was acquired	*Survey of public central banks' official documents, acts, and financial institutions reports available on official web pages.*
Data format	*Raw and analyzed data.*
Experimental factors	*Data described is a survey. There was not a random assignment component in data.*
Experimental features	*Countries have been selected considering the availability of data. For the selected OECD and non-OECD countries, the central bank's website and official documents therein, have been consulted to retrieve information on the type of institutional governance, the mandate, the type of green regulation (if any), the institution responsible for its implementation or promotion. The collected information has been used to build the dataset.*
Data source location	*The data were analyzed at the Scuola Superiore Sant’Anna (Pisa, Italy) and Ruhr University Bochum (Bochum, Germany)*
Data accessibility	https://doi.org/10.17632/9s5pt9dddn.1
Related research articles	*D'Orazio, P. and Popoyan, L. (*2019*)****Fostering green investments and tackling climate-related financial risks: Which role for macroprudential policies?,****Ecological Economics, Volume 160, June*2019*, Pages 25–37**[3]* *D'Orazio, P. and Popoyan, L. (*2019*)****Central banks and green prudential regulation: State-of-the-art, challenges and perspectives****, Working Paper.**[4]*

**Table:** 

**Value of the data** • The dataset described in this article is of use for the scientific community as it is the first centralized collection of public data on the usage of green macroprudential tools, their juridical reference, and objective, as well as central banks’ governance type and mandates. • The data described in this article can be used to identify and understand current and future trends, and the role of central banks and regulators, in sustainable finance. • The green macroprudential dataset and statistics presented in this article are critical to understanding the direction and diffusion of greening prudential regulations, and the role of central banks in the low-carbon transition. • It is essential that data on the usage and diffusion of green prudential tools are updated and made available to the researchers, considering the actively evolving debate on the adoption of such instruments and their implementation in different institutional setup worldwide.

## 1 Data

The dataset is a unique source of information regarding the state-of-the-art green prudential regulations and instruments adopted worldwide, together with the data for the governance type and monetary policy goal.

The dataset is built by relying on a comprehensive survey of official documents of national central banks, financial institutions, and international organizations. The investigation ended on October 2018, and it has been updated on February 2019.

The dataset includes 56 countries, of which 31 are members of the OECD and 37 are under the Basel III Accord. The choice regarding the countries to include in the database is related uniquely to the availability of data regarding the adoption, or discussion about the implementation, of green prudential regulations.

The sampled countries are located mostly in Europe (43%), 25% belongs to the Asia and Pacific area, 16% to the American continent, 7% is located in the Middle East and Central Asia, and 9% is composed of African countries.

Regarding the income level (defined according to the World Bank indicators [1] and World Bank countries’ classification by income [2]), 59% of the sample is composed of high-income countries, 21% by lower-middle-income countries, 18% by upper-middle-income countries and finally, by 2% of low-income countries.

**Fig. 1:**
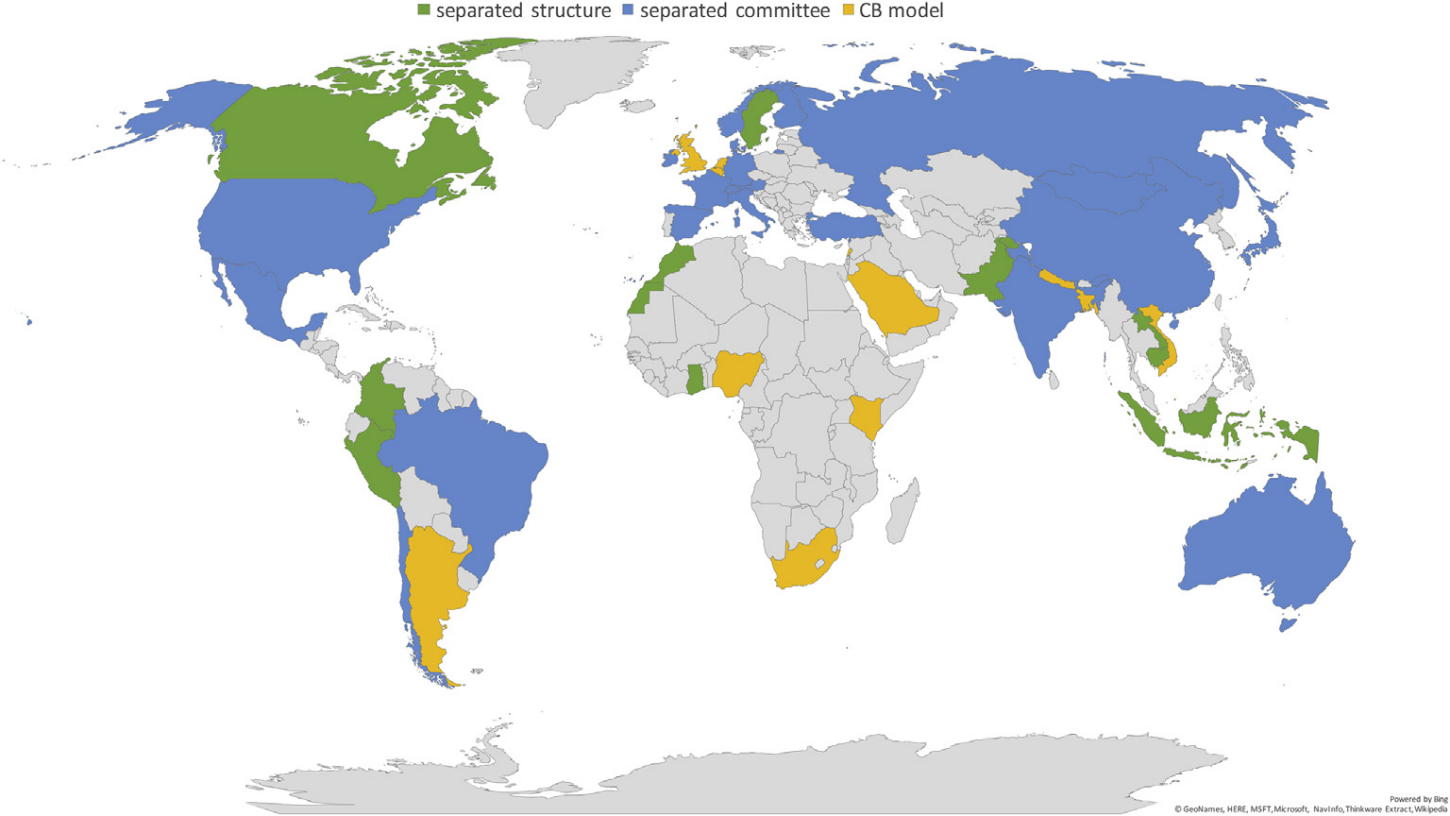
Institutional architectures (CBM index). source: Authors’ elaboration based on the data presented in this article.

**Fig. 2:**
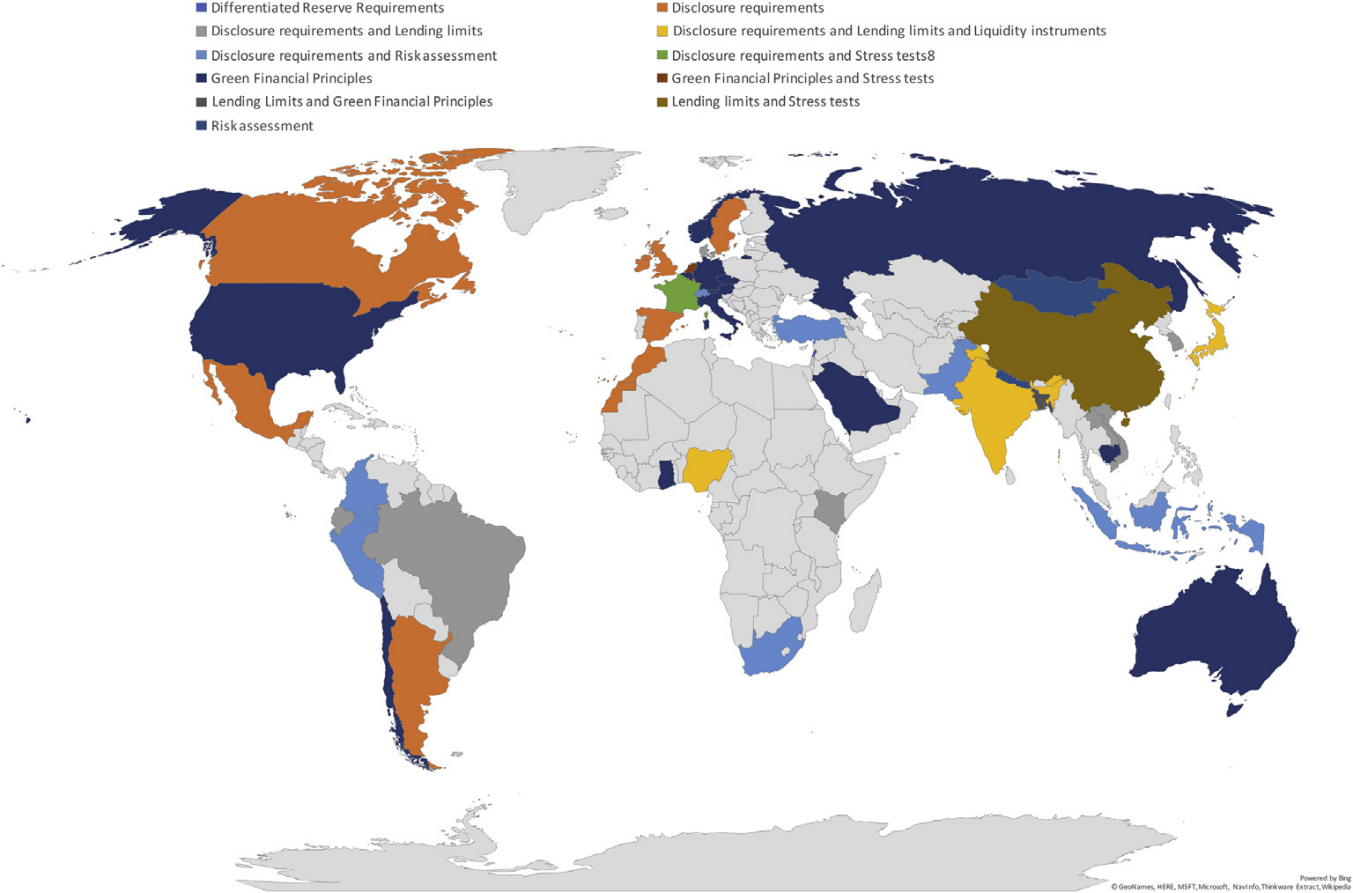
Green prudential regulation index (GPPI). source: Authors’ elaboration based on the data presented in this article.

**Fig. 3:**
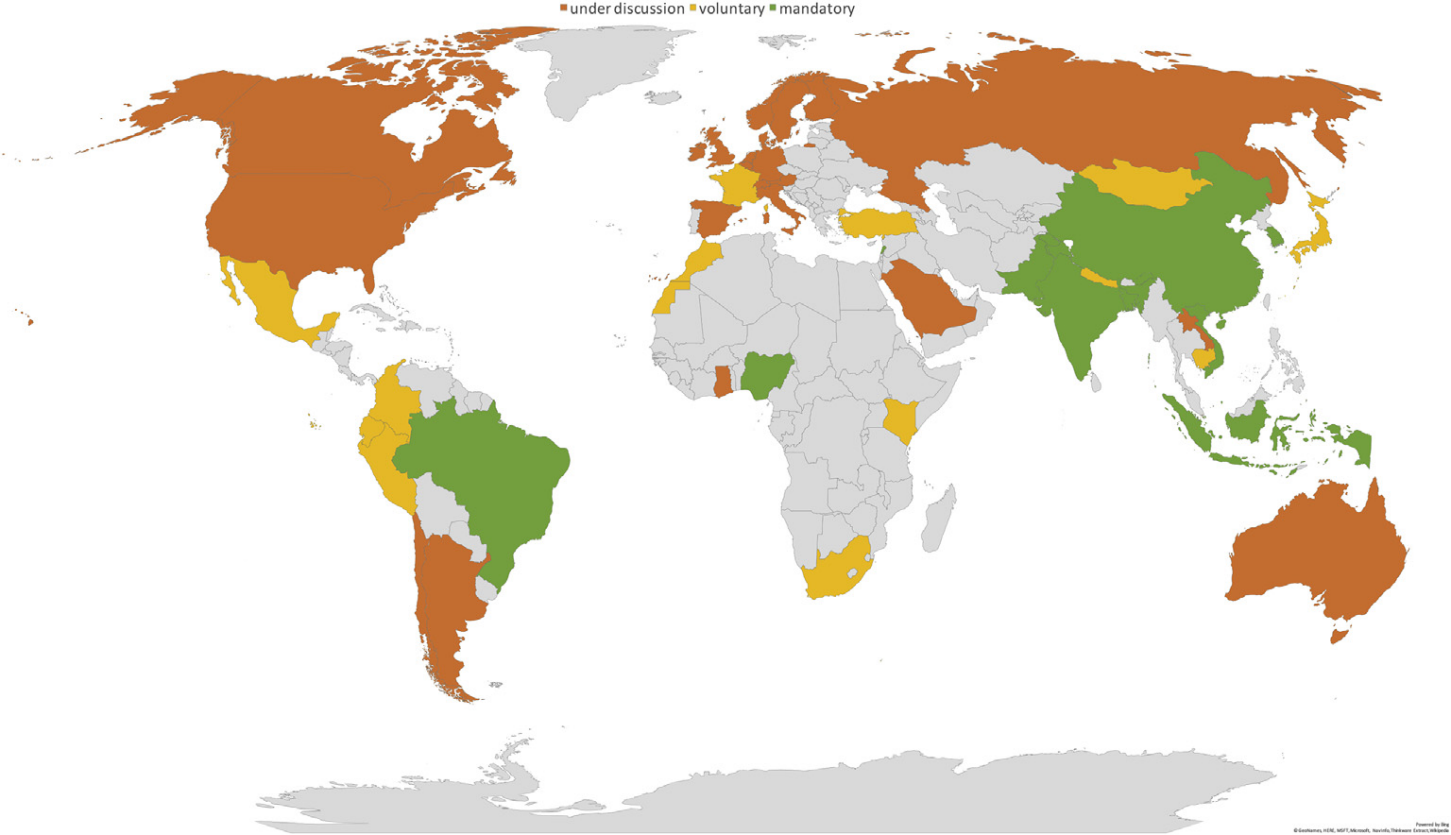
Green macroprudential index (GMI). source: Authors’ elaboration based on the data presented in this article.

A summary of the sample's characteristics, frequencies and shares are provided in Table 1, Table 2, respectively. Fig. 1 offers an overview of the diffusion of the three types of institutional frameworks (i.e., governance types), whereas Fig. 2 shows the worldwide adoption of the different green prudential requirements, sorted according to the Green Prudential Regulation Index we developed in the investigation by relying on the gathered data (for additional details, see Table 2 and Section 2). Fig. 3 presents the distribution of the sampled countries according to whether the adopted (or under discussion) green prudential regulation is binding for the financial sector.

**Table 1:** Characteristics of the sample.

Year of the survey (last update)	February 2019
Sample coverage	56 countries
Members of the Basel Accords	37 countries, 2 observers
OECD members	31 countries

**Table 2:** Composition of the sample: frequencies and shares.

*Statistic*		*Frequency*	*Share*
*By income level*			
	High-income	33	59%
	Upper-middle-income	10	18%
	Lower-middle-income	12	21%
	Low-income	1	2%
*By geographical area*			
	Africa	5	9%
	Americas	9	16%
	Asia and Pacific	14	25%
	Europe	24	43%
	Middle east and Central Asia	4	7%
*By central bank policy goal*			
	Inflation targeting	22	39%
	Inflation targeting & price stability	27	48%
	Growth	1	2%
	Mix	6	11%
*By governance type (CBM index)*			
0	Separate structure model	18	32%
1	Separate committee model	26	47%
2	Central bank model	12	21%
*By Green Macroprudential Index*			
0	Mandatory green regulation	10	21%
1	Voluntary green regulation	13	27%
2	Green regulation under discussion at the policy level	25	52%
*By Green Prudential Regulation Index*			
0	Differentiated Reserve Requirements	1	2%
1	Lending Limits and Green Financial Principles	1	2%
2	Disclosure requirements	9	20%
3	Risk assessment	2	4%
4	Green Financial Principles	14	31%
5	Disclosure requirements and Lending limits and Liquidity instruments	3	7%
6	Disclosure requirements and Risk assessment	7	15%
7	Disclosure requirements and Lending limits	6	13%
8	Disclosure requirements and Stress tests	1	2%
9	Lending limits and Stress tests	1	2%
10	Green Financial Principles and Stress tests	1	2%

The data presented in this article have been used in Ref. [3] to perform a state-of-the-art analysis of the adoption of green regulations and prudential instruments and in Ref. [4] to analyze the state-of-the-art green macroprudential architecture and understand the factors that underlie the choice of particular institutional setups at the country level.

## 2 Experimental design, materials, and methods

The following two main strategies were used to find the relevant information to construct the dataset. First, the keywords “green finance” and “country name” were searched using various combinations to find out the majority of data sources. The selected keywords were combined with one or more of the following terms: “macroprudential policy,” “central bank,” “climate change,” “sustainable.” The keywords’ search was then further improved by specifying, for example, the regulatory authority, if any, and the name of the central bank. The second strategy consisted in first, to identify the institutions which are known to be reporting various initiatives in sustainable finance, and second, to examine their websites.

The collected data were used to build the Green Macroprudential Index (GMI) and the Green Prudential Instrument Index (GPII).

The **GMI** has been created by setting up three categories; namely (i) **under discussion**, to which it is assigned an index equal to zero (GMI = 0) and defines countries that are currently discussing the possibility of introducing green regulations, (ii) **voluntary**, to which is assigned an index equal to one (GMI = 1) and defines countries that developed a voluntary green regulation; and, finally (iii) **mandatory**, to which it is assigned the index equal to 2 (GMI = 2), and defines countries that adopted a green prudential regulation which is already in force. The **GPII** is composed of 11 categories, as shown in Table 2.

Both the GMI and GPPI indices are particularly worth as they can be used to offer a clear picture of different types of currently available green macroprudential regulations, binding conditions for countries, together with their geographical adoption and diffusion.

An additional index, the CBM, has been constructed in order to present the distribution of the different governance architectures. The index is as follows; it takes value of CBM = 0 in the case of the separate structure model, CBM = 1 in the case of the separate committee model and CBM = 2 in the case of the central bank model.
