# Supplementary material for: Dataset on green macroprudential regulations and instruments: Objectives, implementation and geographical diffusion
Source: Data Brief. 2019 Mar 21;24:103870. doi: 10.1016/j.dib.2019.103870 (PMC6446131; doi:10.1016/j.dib.2019.103870)
Supplement: Multimedia component 1 [file mmc1.pdf]

Conflict of Interest and Authorship Conformation Form

- All authors have participated in (a) conception and design, or analysis and interpretation of the data; (b) drafting the article or revising it critically for important intellectual content; and (c) approval of the final version.

- This manuscript has not been submitted to, nor is under review at, another journal or other publishing venue.

- The authors have no affiliation with any organization with a direct or indirect financial interest in the subject matter discussed in the manuscript

Bochum, 18th February 2019

Paola D’Orazio

Lilit Popoyan
